# Linear and Nonlinear Rheology Combined with Dielectric Spectroscopy of Hybrid Polymer Nanocomposites for Semiconductive Applications

**DOI:** 10.3390/nano7020023

**Published:** 2017-01-24

**Authors:** Roland Kádár, Mahdi Abbasi, Roxana Figuli, Mikael Rigdahl, Manfred Wilhelm

**Affiliations:** 1 Chalmers University of Technology, Department of Materials and Manufacturing Technology, SE-41296 Gothenburg, Sweden; mikael.rigdahl@chalmers.se; 2 Karlsruhe Institute of Technology (KIT), Institute for Polymer Technology and Polymer Chemistry, DE-76131 Karlsruhe, Germany; mahdi.abbasi@partner.kit.edu (M.A.); roxana.figuli@kit.edu (R.F.); manfred.wilhelm@kit.edu (M.W.)

**Keywords:** polymer nanocomposites, graphite nanoplatelets, carbon black, electrical conductivity, shear rheology, extensional rheology

## Abstract

The linear and nonlinear oscillatory shear, extensional and combined rheology-dielectric spectroscopy of hybrid polymer nanocomposites for semiconductive applications were investigated in this study. The main focus was the influence of processing conditions on percolated poly(ethylene-butyl acrylate) (EBA) nanocomposite hybrids containing graphite nanoplatelets (GnP) and carbon black (CB). The rheological response of the samples was interpreted in terms of dispersion properties, filler distortion from processing, filler percolation, as well as the filler orientation and distribution dynamics inside the matrix. Evidence of the influence of dispersion properties was found in linear viscoelastic dynamic frequency sweeps, while the percolation of the nanocomposites was detected in nonlinearities developed in dynamic strain sweeps. Using extensional rheology, hybrid samples with better dispersion properties lead to a more pronounced strain hardening behavior, while samples with a higher volume percentage of fillers caused a drastic reduction in strain hardening. The rheo-dielectric time-dependent response showed that in the case of nanocomposites containing only GnP, the orientation dynamics leads to non-conductive samples. However, in the case of hybrids, the orientation of the GnP could be offset by the dispersing of the CB to bridge the nanoplatelets. The results were interpreted in the framework of a dual PE-BA model, where the fillers would be concentrated mainly in the BA regions. Furthermore, better dispersed hybrids obtained using mixing screws at the expense of filler distortion via extrusion processing history were emphasized through the rheo-dielectric tests.

## 1. Introduction

Graphene stands out as potential nanofiller in polymeric melts due to its outstanding mechanical, dielectric, barrier, thermal, etc., properties [1]. However, significant challenges remain to be overcome in the development of graphene-based consumer products. In this framework, of particular importance is understanding the flow field-matrix-filler interaction in polymer nanocomposites [2,3]. By the method of processing, the microstructure can be tailored to attain the desired material properties by de-agglomerating the particles, improving dispersion and ensuring the desired orientation of the nanofillers in the melt [4]. Due to the large/fast deformations employed, polymers are subjected to nonlinear deformations during processing, with the interactions between the flow field, e.g., extrusion flow, matrix and fillers dictating the overall microstructure dynamics and subsequent material properties/performance. Therefore, it is important to probe material linear, as well as nonlinear rheological properties in simple configurations and to investigate the associated microstructural effects and their relationship with the materials’ mechanical, electrical, barrier, etc., properties.

Rheology is an important tool to characterize polymer nanocomposites in relation to understanding the underlying complex matrix-filler and filler-filler interactions and for the design of processing operations, e.g., see [5,6,7,8,9,10], among many others. In particular, Fourier-transform rheology has been used to determine the percolation threshold, morphological changes and microstructural interactions in percolated systems, the influence of various particle characteristics and modeling the rheological material response [11,12,13,14,15]. Changes in the strain amplitude-dependent nonlinearities’ scaling were reported for percolated systems, including a maximum peak in the third relative higher harmonic of the polycaprolactone/multi-walled carbon nanotube (PCL/MWCNT, 1D thread shape), polycaprolactone/organomodified montmorillonite (PCL/OMMT, 2D plate shape with high aspect ratio) and the polyethylene/multi-walled carbon nanotube (PE/MWCNT), but not for polycaprolactone/precipitated calcium carbonate (PCL/PCC, 3D cubic shape) [14,15]. The nonlinear rheological behavior of ethylene butyl acrylate (EBA)-carbon black (CB) composites was investigated by Leblanc and Jäger [13]. Strongly nonlinear viscoelastic properties were observed, as well as an unusual complexity of carbon black influence on their viscoelastic response. Furthermore, a dual morphology composed of PE-rich crystallites and CB-rich amorphous butyl acrylate (BA) regions was proposed. It is known that, among different substances used in composites, carbon black is unique in its ability to significantly enhance the properties while lowering the cost [16]. However, applications such as semi-conductive layers for high voltage cables require highly filled carbon black nanocomposites that can pose problems to the manufacturing of the cables [17,18]. Hybrid EBA-GnP-CB nanocomposites for semiconductive high voltage cable applications were considered by Arino et al. [3] and took advantage of synergistic effects due to the particle shape of the two fillers. The electrical conductivity of the nanocomposites was optimized via melt extrusion with different processing histories with a strong influence on the electrical percolation behavior [3].

The nanoparticle network in nanocomposites could strongly be affected by nonlinear deformations, e.g., large amplitude oscillatory shear (LAOS) or extensional flows, which might result in network failure under large deformations. Extensional properties of nanocomposites, e.g., strain hardening, are affected not only by the stretching and orientation of the polymer chain matrix, but also by the interactions between polymer-particle and particle-particle. Hassanabadi et al. [12] investigated the effect of the shape of particles in nanocomposites and concluded that linear and nonlinear rheological properties of nanocomposites containing platelet particles, e.g., clay at concentrations higher than 2.5 wt %, are dominated by particle nanonetworks over polymeric chains’ contribution, while those containing spherical particles, e.g., ${\mathrm{CaCO}}_{3}$, behave similar to polymer melts. Sinha Ray and Okamoto [19] reported a deviation from the Trouton ratio, $\eta_{E}=3\eta$, for the shear and extensional properties of polymer layered silicate nanocomposites in the range of the linear viscoelastic regime.

The combination of rheological measurements with a second characterization method, e.g., dielectric spectroscopy, was developed in order to gain unique information about molecular dynamics and the structure of time- and shear-dependent phenomena [20,21,22,23,24]. Purely rheological methods provide only information on the macroscopic behavior of the samples. This means that rheology can only be used to determine macroscopic and averaged material functions. Obtaining simultaneously additional information on the microstructure or even molecular level structure is often needed for a better understanding of the rheological behavior. Thus, dielectric spectroscopy has proven to be a very useful tool for studying the structure and the dynamics of polymeric systems [20]. This knowledge is very important for the development of new materials for industrial applications, with specific electrical properties [21,23,25]. Generally, the conductivity of a polymer, rather than being a linear function of the concentration of the added particles, is almost insensitive for lower concentrations and rises abruptly as the electrical percolation threshold is reached. This occurs at a critical concentration where the particles get in contact with each other, and as a consequence, a continuous electrical path of the doped particles is built throughout the polymer matrix. That is, when the filler content is low, the mean distance between conducting particles is large, and the conductivity is restricted by the presence of the insulating matrix; but by increasing the conductive phase content, the conducting particles get closer, and at that critical concentration the electrical properties are dominated by them [24,26].

### Present Study

In the present study, the influence of processing conditions on percolated ethylene butyl acrylate (EBA) nanocomposites containing graphite nanoplatelets (GnP) and GnP-carbon black (CB) was investigated from the rheological point of view. Thermogravimetric analysis, scanning electron microscopy, optical microscopy and image analysis, X-ray diffraction, mechanical properties and capillary viscometry (high shear rates) analysis of the samples used in the present study can be found in Arino et al. [3], and the two studies should be interpreted complementarily. In this publication, dynamic frequency and strain sweeps, extensional and rheo-dielectric tests are presented. Overall, the results help with correlating the nonlinear, dielectric and extensional rheological measurements to the influence of the extrusion processing conditions of GnP-CB-EBA hybrid nanocomposites and, thus, improve the understanding of process and material design, for optimal final product properties.

## 2. Materials and Methods

### 2.1. Materials and Preparation

The nanocomposites analyzed were composed of poly(ethylene-butyl acrylate), EBA, as the matrix, graphite nanoplatelets, GnP, and carbon black, CB. The EBA matrix contained 17 wt % butyl acrylate (BA) and had a melting temperature of 100 °C and a density of 0.925 g/${\mathrm{cm}}^{3}$ [18]. The CB was a medium-structured carbon black (CB) ENSACO^®^ 260G, TIMCAL Graphite and Carbon, Bodio, Switzerland, and the GnP used was xGnP Grade M5 from XG Sciences, Lansing, MI, USA. The CB was characterized by a surface area of 70 $m^{2}$/g and a density of 1.8 g/${\mathrm{cm}}^{3}$, while the GnP had a thickness of 6–8 nm, a characteristic diameter of 5 μm, a surface area of 120–150 $m^{2}$/g and a density of 2.2 g/${\mathrm{cm}}^{3}$ (manufacturer data). The composition of the EBA-GnP-CB hybrid nanocomposites was optimized by Oxfall et al. [2], and Arino et al. [3] further improved the electrical properties of the nanocomposites by varying the processing conditions, such as screw geometry, temperature profile and screw speed. A Brabender, Brabender GmbH, Duisburg, Germany, 19/25D (barrel diameter of 19 mm and barrel length of 19×25) single-screw extruder, equipped with a 1.5-mm radius circular die was used for sample preparation. More details on the compounding of the master batches can be found in [3].

**Figure 1:**
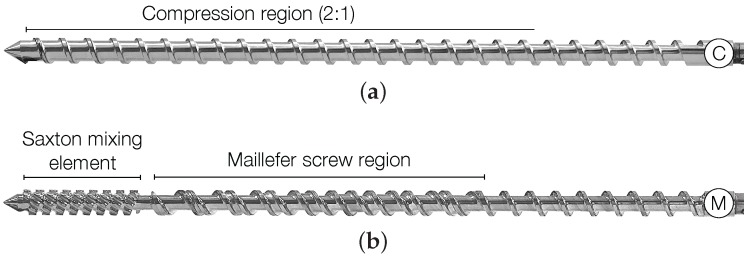
The screw types used to control the deformation history of the nanocomposites analyzed using a Brabender 19/25D single-screw extruder: (**a**) C-screw, conventional geometry with 2:1 compression ratio; and (**b**) M-screw, distributive mixing screw composed of a Maillefer region a Saxton mixing element.

Two screw geometries were used for sample preparation: Figure 1a, the C-screw, a conventional screw with a compression ratio 2:1; and Figure 1b, the M-screw, a distributive mixing screw comprising a Maillefer region and a Saxton mixing element. As the processing parameters, two screw speeds of 50 rpm and 100 rpm and two die temperatures, 160 °C and 180 °C, both die temperatures following a progressive increase in temperature along the barrel, were used [3].

Samples optimized by Arino et al. [3] were pelletized and then compression molded into disks of 0.9–1.2 mm in thickness and 25 mm in diameter for rotational rheometry and into rectangular specimens of 20 mm in length, 10 mm in width and approximately 1 mm in thickness for extensional tests. In addition, the EBA and EBA-GnP nanocomposites were used as reference samples. The filler concentration corresponds to the percolation in the respective compositions [3]. A list of the investigated samples, their composition and processing conditions are listed in Table 1, namely the die temperature, $-T_{p}$, screw rotational speed, *n*, apparent shear rate inside the die, ${\dot{\gamma }}_{a}$, characteristic relaxation time, λ, and the resulting processing Weissenberg numbers, $Wi=\lambda {\dot{\gamma }}_{a}$ as the control parameter [27]. The apparent shear rate in the die was computed as:

(1) $${\dot{\gamma }}_{a}=\frac{4Q}{\pi R^{3}}$$

where *Q* is the flow rate inside the die obtained from applying the mass conservation law between the barrel/screw zones and the die and *R* is the die radius. The characteristic polymer relaxation time, λ, was defined as the inverse of the crossing point between the dynamic moduli in a dynamic frequency sweep test.

### 2.2. Rheological Characterization

**Figure 2:**
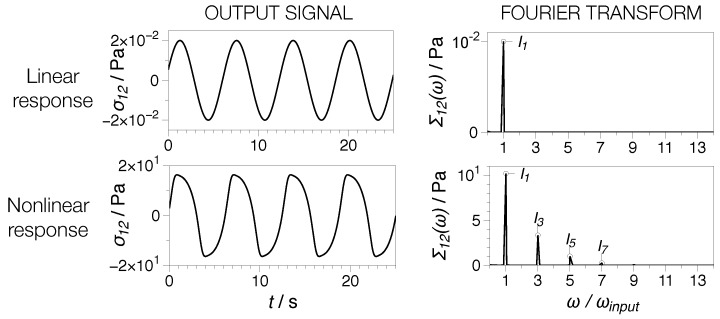
Generic principle of Fourier transform rheology showing time-dependent linear and nonlinear stress material response to a sinusoidal strain input and corresponding Fourier transform of the output signal showing the presence of higher harmonics in the nonlinear case.

**Figure 3:**
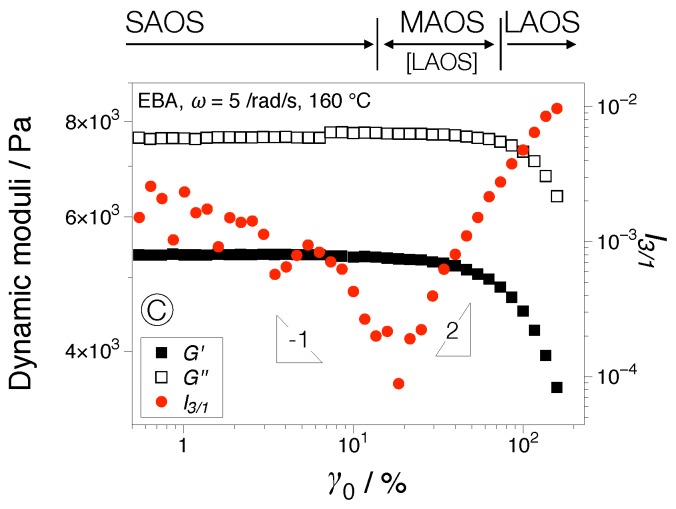
Dynamic strain sweep (ω=5 rad/s) comparing the dynamic moduli, $G^{\prime }$ and $G^{\prime \prime }$, and the relative first higher harmonic, $I_{3/1}$, for poly(ethylene-butyl acrylate) (EBA) at 160 °C.

The rheological characterization was performed on the TA Instruments ARES G2, New Castle, DE, USA, Anton Paar MCR702 TwinDrive, Graz, Austria, (dynamic frequency sweeps) and TA Instruments ARES (dynamic strain sweeps , extensional and rheo-dielectric experiments), New Castle, DE, USA, rheometers [28]. Plate-plate 25-mm geometries were used, with gap setting according to the disk thickness. The extensional measurements were performed using the extensional viscosity fixture (EVF, TA Instruments) New Castle, DE, USA. All rheological tests were performed at 160 °C. Oscillatory shear experiments were performed in the linear and nonlinear regime, i.e., small amplitude oscillatory shear (SAOS) and large amplitude oscillatory shear (LAOS) [29]. For nonlinear analysis, Fourier-transform (FT) rheology was used, an advanced rheological characterization method [29]. In an SAOS test, a sinusoidal input strain signal results in a sinusoidal material response whose Fourier spectrum is characterized by one characteristic peak corresponding to $\omega /\omega_{i}=1$, where $\omega_{i}$ for the input angular frequency; see Figure 2. In this regime, the the material functions describing the materials’ response are the dynamic moduli, i.e., the storage, $G^{\prime }$ and loss $G^{\prime \prime }$ modulus [30]. In an LAOS test, the sinusoidal input strain signal results in a nonlinear material response whose Fourier spectrum is in turn characterized by one characteristic peak corresponding to $\omega /\omega_{i}=1$ complemented by higher harmonics; see Figure 2. The nonlinear signal represented in the figure was obtained using a 5-mode Giesekus model [31]. Thus, the nonlinear shear material stress response in FT rheology can be written as [29,32]:

(2) $$\sigma_{12}\left(t\right)=I_{1}\sin\left(\omega t+\delta_{1}\right)+I_{3}\sin\left(3\omega t+\delta_{3}\right)+I_{5}\sin\left(5\omega t+\delta_{5}\right)...$$

where $\sigma_{12}$ is the shear component of the stress tensor, $I_{n}$ are the (odd) intensities of the harmonics and $\delta_{n}$ the corresponding phase angles. The third relative higher harmonic, $I_{3/1}$, is generally taken as a measure of the nonlinearities in the signal, as it holds the dominant nonlinear contribution to the signal. In contrast to linear viscoelastic experiments, nonlinear tests are more sensitive to the molecular make-up of the materials, while at the same time being subjected to nonlinear conditions closer to processing flows. A commonly-used derived material nonlinear parameter, the *Q*-parameter, is defined as [33]:

(3) $$Q=\frac{I_{3/1}}{\gamma_{0}^{2}}$$

where $\gamma_{0}$ is the applied strain amplitude. An example of a dynamic strain sweep test using the EBA matrix comparing the dynamic moduli and the third relative higher harmonic, $I_{3/1}$ is shown in Figure 3. At low strain amplitudes (SAOS), the dynamic moduli are independent of the applied strain amplitude, while instrumentation noise characterizes the $I_{3/1}$ behavior, $I_{3/1}\propto \gamma_{0}^{-1}$. Generically, the limit of the linear regime (SAOS) is where the dynamic moduli are no longer independent of the applied strain amplitude. A more accurate change in material response is recorded via an increase in $I_{3/1}$. At the onset of the nonlinear regime, $I_{3/1}\propto \gamma_{0}^{2}$, the region that is referred to as medium amplitude oscillatory shear (MAOS) [33] or intrinsic LAOS [34]. Thereafter, the LAOS regime is achieved, with $I_{3/1}$ expected to level off at the high strain amplitudes. One of the major benefits of Fourier-transform (FT) rheology is the improvement in sensitivity, as can be seen when comparing the dynamic moduli varying within the same order of magnitude compared to $I_{3/1}$ varying within two orders of magnitude; Figure 3.

### 2.3. Extensional Rheology and the Molecular Stress Function Theory

The extensional rheological properties of EBA, as well as their composites were analyzed using the molecular stress function (MSF) constitutive equation. This model predicts the strain hardening in extensional flows using two nonlinear parameters, β and $f_{\max}$ [35]. Constant β and $f_{\max}$ govern the slope of strain hardening and the steady-state value of the stress growth coefficient, $\eta_{E,\;\max}^{+}$, in extensional flows. Here, a simplified time-strain separable version of the MSF model was used, which was presented by Abbasi et al. [36] for hyper-branched structures under extension and LAOS deformation. This constitutive equation was verified for EVA nanocomposites with different particle shapes [37]:

(4) $$\sigma \left(t\right)=\sum_{i=1}^{N}\int_{-\infty }^{t}\frac{g_{i}}{\tau_{i}}e^{\frac{\left(t-t^{\prime }\right)}{\tau_{i}}}f^{2}\left(t,t^{\prime }\right)\;S_{DE}^{IA}\left(t,t^{\prime }\right)dt^{\prime }$$

(5) $$S_{DE}^{IA}=5S=5\left[\left(\frac{1}{J-1}\right)B-\left(\frac{1}{\left(J-1{\left(\mathrm{tr}C+13/4\right)}^{0.5}\right)}\right)C\right]$$

(6) $$J=\mathrm{tr}B+2{\left(\mathrm{tr}C+13/4\right)}^{0.5}$$

(7) $$\frac{\partial f^{2}}{\partial \epsilon }=\frac{1}{2}\beta f\left(S_{11}-S_{22}-\frac{f^{2}-1}{f_{max}^{2}-1}\sqrt{S_{11}+0.5S_{22}}\right).\;$$

Elements in bold, σ, S, B and C, are the stress, measure of strain, Finger and Cauchy tensors, respectively. Relaxation spectra $g_{i}$ and $\tau_{i}$ were obtained by fitting the Maxwell model on $G^{\prime }$ and $G^{\prime \prime }$ data. The stretching function, *f*, shows the backbone stretch, which causes the strain hardening phenomenon in the polymer chains and is a function of Hencky strain, ϵ, and nonlinear parameters β and $f_{\max}^{2}$. Equations (4)–(7) represent an integral time-strain separable constitutive equation, which was fitted to the uniaxial stress growth coefficient data by adjusting the β and $f_{\max}$ as fitting parameters. These parameters were used as a bridge between the strain hardening criteria and the polymer chain and particle network in nanocomposites.

### 2.4. Combined Rheology: Dielectric Spectroscopy

**Figure 4:**
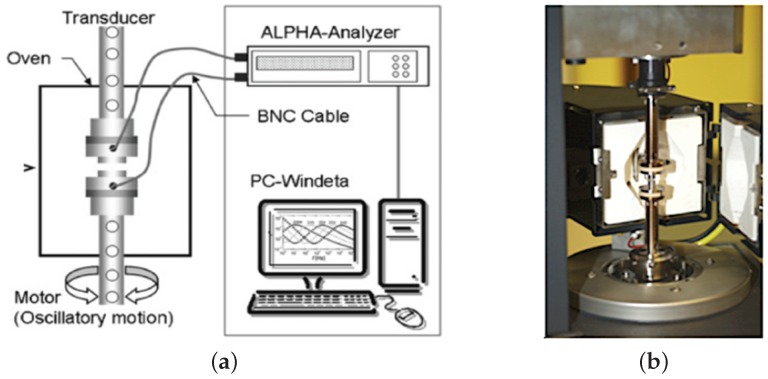
Simultaneous rheo-dielectric measurements performed to characterize the EBA nanocomposites. (**a**) Diagram of the combined rheometer and dielectric analyzer for determining the raw stress data and the dielectric spectra in situ. (**b**) Image of the rheo-dielectric geometries.

Experimental data were analyzed by dielectric permittivity vs. frequency formalism. Dielectric spectroscopy (DES) is a dynamic technique quantifying the molecular dynamics and conductivity processes in dielectric (insulating or semiconducting) materials due to their interaction with electromagnetic fields. In a linear system, when an external AC electric field is applied to a dielectric medium with a permanent dipole moment, the complex dielectric function can be expressed as follows:

(8) $$\epsilon *\left(f_{e}\right)=\epsilon^{\prime }\left(f_{e}\right)-i\epsilon^{\prime \prime }\left(f_{e}\right)$$

where $\epsilon^{\prime }\left(f_{e}\right)$ and $\epsilon^{\prime \prime }\left(f_{e}\right)$ are the real and imaginary parts. The dielectric function $\epsilon *(f_{e})$ describes the dielectric behavior of the material and contains information about the molecular transport and relaxation processes by monitoring the charged ions and electric dipoles existing in the molecule. In general, transport processes in solids, especially in complex structured polymers, depend on their internal morphology, as well as on the influence of important physical properties. The dielectric function $\epsilon *(f_{e})$ depends on several variables, such as temperature, frequency, the molecular mobility within the material, the macroscopic orientation of the polymer chains, electromagnetic fields and the applied mechanical loads, including pressure and tensile stresses [38]. The viscoelastic properties of the chain reflect a particular average of the chain conformation, i.e., the isochronal orientational anisotropy of individual entanglement segments. The dielectric property reflects the orientational correlation of two segments at two separate times (e.g., $t_{0}=0$ and $t_{1}=\tau$) and can be converted to the motion and fluctuations of the molecules of the dipole arrangement with respect to the molecular axis. This means that the chain conformations and motions are differently averaged in the viscoelastic and dielectric properties. Therefore, the combination of rheological and dielectric properties (rheo-dielectric properties) can give detailed information about the relation between local molecular relaxation dynamics and macroscopic response to mechanical forces. As well as the chain dynamics, the rheo-dielectric combination might be very useful to investigate various materials and shear-induced changes. For the investigation of short length scale dynamic measurements, the most prominent development in the field of combined techniques is rheo-dielectric, as can be seen in Figure 4. The rheo-dielectric measurements were performed in the linear and nonlinear viscoelastic regimes at a strain amplitude of $\gamma_{0}=1\%$ and an angular frequency of ω=0.5 rad/s and $\gamma_{0}=100\%$, ω=1 rad/s, respectively. For the rheo-dielectric measurements, the sample was placed between two custom-made INVAR steel parallel plate electrodes of 25 mm in diameter and a spacing between of 0.9 and 1.5 mm. Therefore, the capacitance of the empty cell was between 15 and 7 pF. The accuracy of the rheometer gap control, which defines the spacing between the electrodes, was in the order of ±0.5μm. Dielectric measurements were carried out with an ALPHA analyzer (Novocontrol, Hundsangen, Germany) using a custom-made experimental setup [39,40]. The broadband dielectric measurements were carried out within the frequency ($f_{e}$) range $10^{0}$ Hz–$10^{7}$ Hz. The resolution of the setup in tanδ for the applied experimental conditions was approximately $\Delta \tan\delta \approx 10^{-5}$ [41].

## 3. Results and Discussion

### 3.1. Linear and Nonlinear Oscillatory Shear

**Figure 5:**
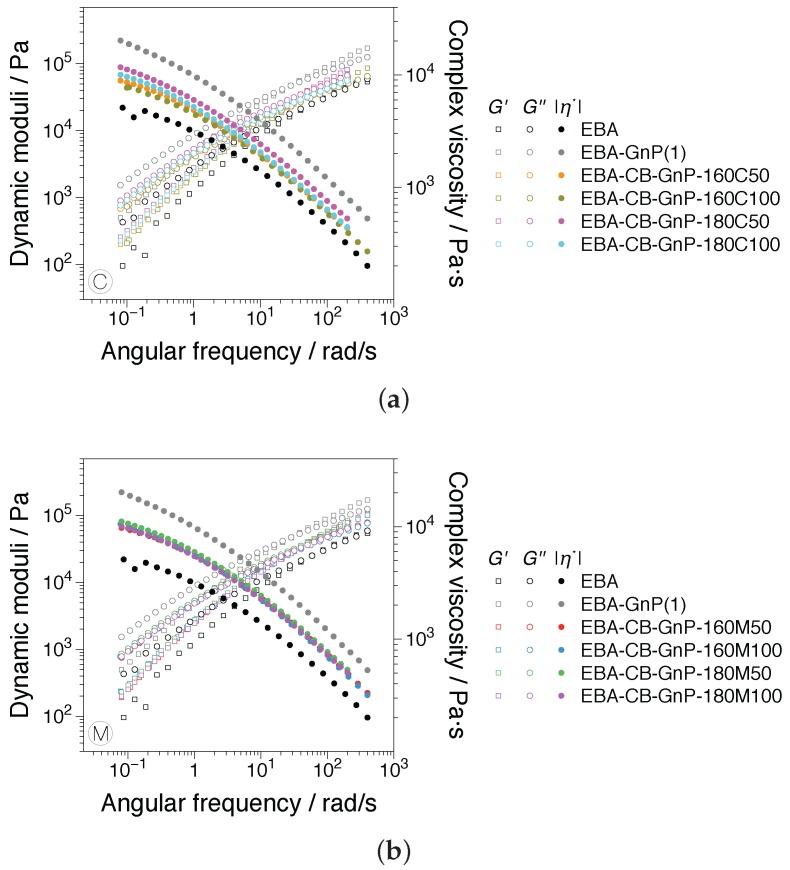
Dynamic frequency sweep measurements in the linear viscoelastic regime of the samples investigated, prepared using (**a**) the conventional screw, C, and (**b**) the distributive mixing screw, M. In both diagrams, the EBA matrix and EBA-GnP are plotted as references.

**Figure 6:**
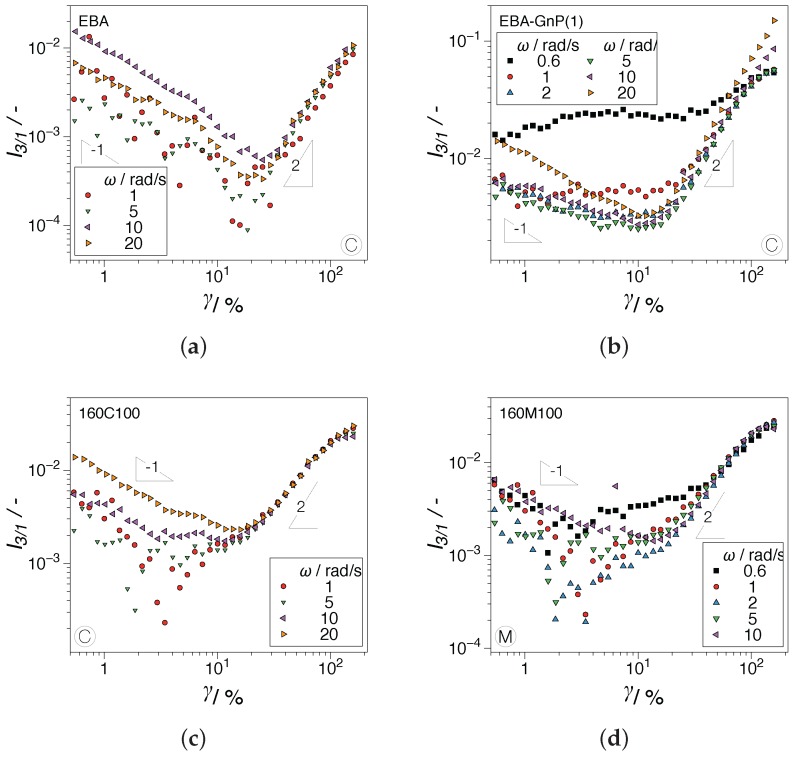
Strain amplitude dependence of the third relative higher harmonic, $I_{3/1}$, at various angular frequencies for: (**a**) EBA, (**b**) EBA-GnP, (**c**) 160C100 and (**d**) 160M100.

Linear viscoelastic dynamic frequency sweeps of the samples investigated are presented in Figure 5. The influence of the processing history is readily observable: the complex viscosity function and corresponding dynamic moduli obtained using the conventional compression screw, C, Figure 5a, with a standard deviation up to 1200 Pa·s, show increased scattering between the hybrid nanocomposites (all containing 5 vol % of filler and the same GnP/CB composition), compared to the M screw, Figure 5b, with a standard deviation up to 430 Pa·s. The contrast can be due to a better distribution of the fillers expected for the mixing screw M. This interpretation is consistent with the optical micrograph image analysis performed by Arino et al. [3]. Their analysis showed up to a 50% decrease in the length of nanoplatelets/agglomerates in the M-screw samples when compared to the C-screw samples, in the 10–30 μm size distribution prevalent in the analysis. Thus, a higher amount of agglomerates was identified using a conventional compression screw, the C-screw. As expected, an increase in the rheological material functions is recorded for the nanocomposites with increasing volume fraction when compared with the EBA matrix, with the highest values being recorded for EBA-GnP having the highest amount of filler content (7 vol %). It should be noted that in the measurement dynamic range ($\omega_{\min}=10^{-1}\;\mathrm{rad}/s$), the percolations of the filled systems cannot be observed as a plateau in the storage modulus, $G^{\prime }$. Linear viscoelastic measurements showing the existence of the $G^{\prime }$ plateau in EBA-CB-GnP systems including the studied filler fractions can be found in Oxfall et al. [2]. However, the existence of a rheologically-percolated network can be identified using the increased sensitivity of the nonlinear rheological response of the materials. The strain amplitude dependence of the relative third higher harmonic, $I_{3/1}$, for selected samples is presented in Figure 6. The features outlined using Figure 3 and the comments thereof can be readily observed in the extended angular frequency range and for the nanocomposite measurements. A change in the scaling behavior of the nonlinearities for filled samples can be distinguished similar to the results by Lim et al. [15] and Ahirwal et al. [14]. In addition, and perhaps the most striking difference, the nanocomposites show a different SAOS-MAOS transition in $I_{3/1}$ through the existence of a transition plateau; see Figure 6b–d.

Plateaus were recorded in all filled samples and for all processing conditions, with no distinguishable difference due to their processing history. The nonlinearities at the SAOS-MAOS transition were higher than those recorded for the unfilled polymer, $I_{3/1}<10^{-3}$, with similar values at comparable angular frequencies for the hybrids, $I_{3/1}\approx 1.1\times 10^{-3}$ for ω=1 rad/s, and as high as $I_{3/1}<1.1\times 10^{-2}$ for ω=0.6 rad/s for the GnP samples. Given that the nanocomposite samples investigated have filler compositions above the percolation threshold, the plateaus can be interpreted as evidence of this. It is important to note that the differences in the SAOS-MAOS transition for the nanocomposites can be made at angular frequencies as high as ω=1 rad/s or even higher when compared to the sharp transition observed in the EBA matrix. Thus, nonlinearities are able to indicate the percolation in dynamic ranges where linear viscoelastic measurements, e.g., see Figure 5, cannot emphasize it.

### 3.2. Extensional Rheology

**Figure 7:**
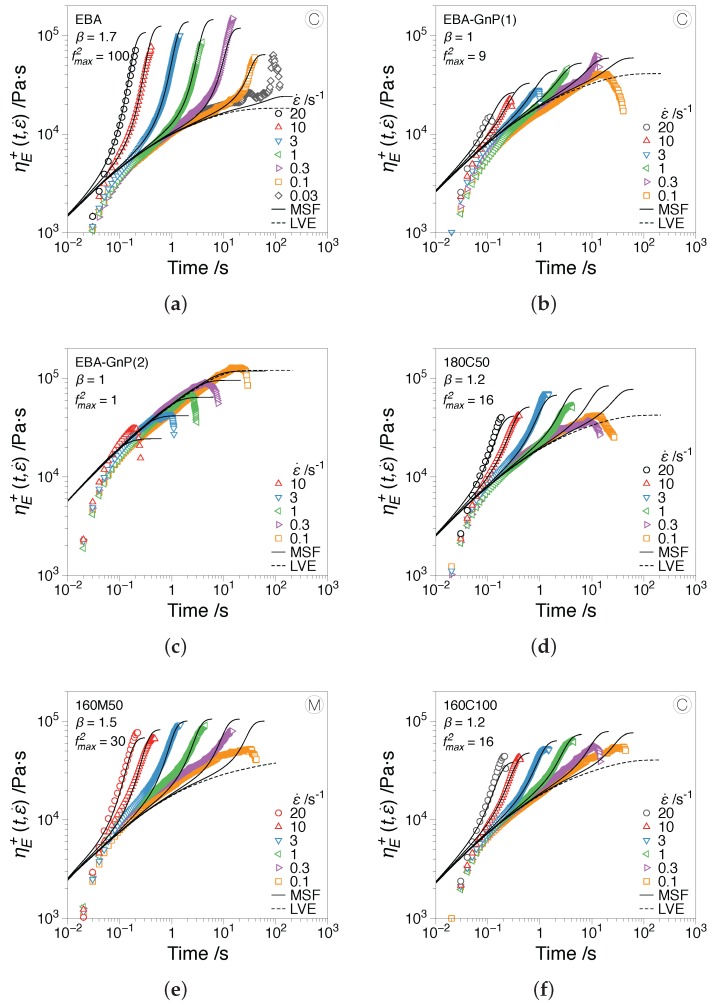
Extensional measurements (symbols) and the MSF theory predictions (solid lines) for (**a**) EBA, (**b**) EBA-GnP(1), (**c**) EBA-GnP(2), (**d**) 180C50 (**e**) 160M100 and (**f**) 160C100. The EBA-GnP(2) contains 9.5 vol% of GnP and was analyzed in order to test the limits of the strain hardening behavior. The dashed line, LVE, represents the linear viscoelastic envelope.

Figure 7 shows the experimental (symbols) and MSF simulation (solid lines) results of the stress growth coefficient as a function of time at different extensional rates for EBA and its composites at 160 °C. Strain hardening phenomenon in EBA proved the presence of branched topologies in the molecules, which resulted in additional friction during the relaxation period compared to a linear molecule. The study of branched topologies of EBA was beyond the scope of the current paper; however, the MSF parameter β=1.7, for unfilled EBA, being less than two, proves a comb-like architecture rather than a branch on branch or Cayley-tree molecular topology [36,42]. A comparison between all samples indicated that neat EBA in Figure 7a showed the highest strain hardening behavior (β=1.7 and $f_{\max}^{2}=100$) due to the higher mobility of polymer chains compared to filled polymer. In other words, the presence of particles in the polymer matrix confined the chain dynamics, which resulted in a lower maximum stretchability of polymer chains ($f_{\max}$), as well as lower onset of strain hardening (β). Experimental data, as well as MSF fitting parameters (β and $f_{\max}^{2}$) in Figure 7a,b demonstrate that the increasing volume content of GnP drastically decreases the strain hardening. Using a special sample containing 9.5 vol % GnP (20 wt %), EBA-GnP(2), no strain hardening was observed (Figure 7c), but rather, strain softening was induced in polymer chains. Polymer composites, including inorganic fillers, e.g., ${\mathrm{CaCO}}_{3}$ [12], glass fiber [43] or crosslinked (hard) rubbers [44], exhibit weaker strain hardening than their matrix polymers. These findings indicated that linear viscoelastic properties in shear (e.g., $G^{\prime }$ and $G^{\prime \prime }$) or extension (linear viscoelastic envelope, LVE, dashed line, computed from the linear viscoelastic behavior, $\eta_{E}^{+}\equiv 3\eta^{+}$, with the relaxation data defined by fitting the Maxwell model on the SAOS dynamic moduli) increased in the presence of rigid particles, whereas the strain hardening factor in extensional deformations decreased. This suggested that under elongational flow, a heterogeneous deformation (a combination of shear, uniaxial and planar extensional flows) is induced around the hard particles, as they cannot be stretched similar to the matrix [44,45]. This complication in flow field does not affect the predictions of strain hardening in uniaxial extensional simulations using the MSF model; however, compared to the neat EBA, some deviations are obvious between the predictions and extensional experiments of nanocomposites. Figure 7c shows that even increasing the GnP content to 9.5 vol % GnP (20 wt %) induces strain softening behavior, which has been rarely seen in the tensile stress growth coefficient (transient extensional viscosity, $\eta_{E}^{+}$) and most probably happened in the shear stress growth coefficient (transient shear viscosity, $\eta^{+}$) of branched polymers. The strain softening phenomenon in extensional experiments (Figure 7c) demonstrates that the region in which elongational flow exists is significantly reduced. This figure shows that strain softening occurs at large Hencky strains ($\epsilon_{H}>2$) where most part of the matrix (EBA polymer chains) is probably pulled out of the extensional region (middle of the sample, between the EVF drums). Therefore, GnP particles constitute a major phase in this region, and shear flow field around the particles is the dominant deformation media in spite of the externally-imposed extensional field. Figure 7d–f shows that screw type M resulted in higher strain hardening (β = 1.5 and $f_{\max}^{2}$ = 30) compared to the screw type C (β = 1.2 and $f_{\max}^{2}$ = 16) regardless of other processing conditions, such as die temperature and screw speed. This intensive strain hardening was related to a better dispersive mixing caused by Saxton mixing elements in screw type M. As a consequence, a better separation between the plates and higher mobility of particles was achieved. However, this result could also justify that a better dispersive mixing results in a weaker shear field around many small particles rather than a few agglomerated ones.

### 3.3. Time Dependence and Electrical Conductivity

**Figure 8:**
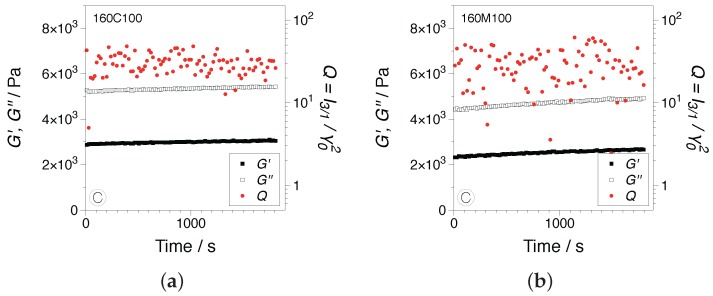
Transient development of the dynamic moduli and the *Q*-parameter in SAOS, γ=1% and ω=0.5 rad/s: (**a**) 160C100 and (**b**) 160M100. All measurements were performed at 160 °C.

Transient dynamic measurements were performed in order to probe the influence of the linear and nonlinear viscoelastic material responses on the morphological and dielectric properties of the samples. Based on the dynamic strain sweep measurements in Figure 6, two experimental conditions corresponding to linear and nonlinear viscoelastic regimes were chosen, namely $\gamma_{0}=1\%$, ω=0.5 rad/s and $\gamma_{0}=100\%$, ω=1 rad/s. Examples of SAOS tests showing the dynamic moduli and the *Q*-parameter, Equation (3) as the nonlinear parameter, are presented in Figure 8. As expected, in the linear regime, no significant influence of the shear deformation was recorded. The dynamic moduli showed a fairly stable development, while the *Q*-parameter falls within the $I_{3/1}$ instrumentation noise region, $I_{3/1}\propto \gamma_{0}^{-1}$, as evidenced by the large scattering in the data.

**Figure 9:**
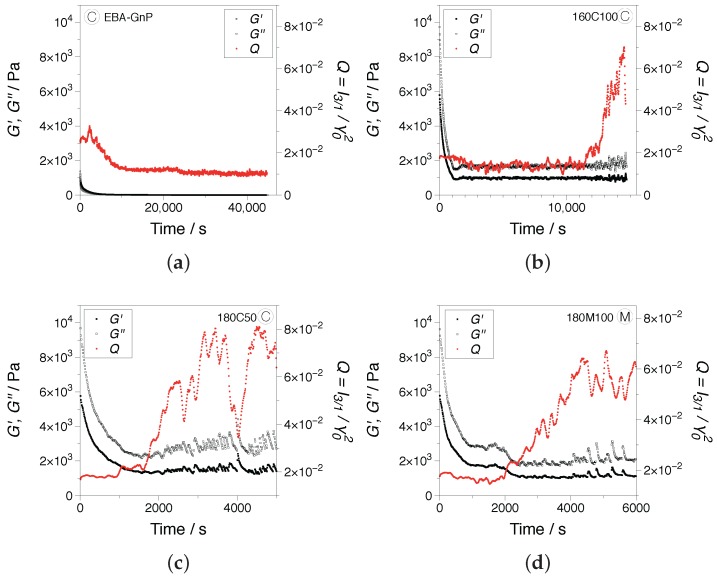
Transient development of the dynamic moduli and the *Q*-parameter in LAOS, γ=100% and ω=1 rad/s, for (**a**) EBA-GnP, (**b**) 160C100, (**c**) 180C50 and (**d**) 180M100. All measurements were performed at 160 °C.

**Figure 10:**
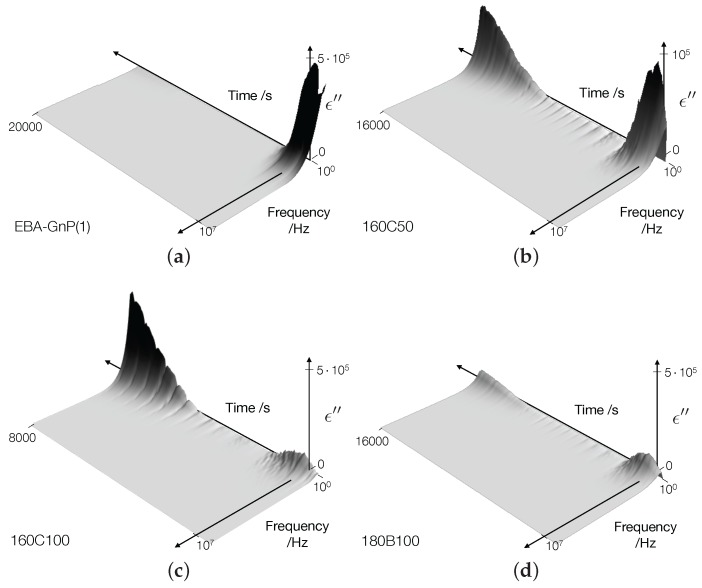
Transient development of the dielectric loss, $\epsilon^{\prime \prime }$, spectra for: (**a**) EBA-GnP, (**b**) 160C50, (**c**) 180C50 and (**d**) 180M100.

Rheo-dielectric measurements were performed in the nonlinear viscoelastic regime; see Figure 9 and Figure 10. At the beginning of the testing time, a drop in the dynamic moduli is being recorded, evidence of the destruction of the existing filler structure in all nanocomposite samples analyzed. In the case of EBA-GnP (Figure 9a), the dynamic moduli and the *Q*-parameter stabilize towards steady state, even after over 40,000 s. At this stage, the magnitude of the *Q*-parameter is below $Q<2\times 10^{-2}$ for all samples. For the hybrids, an increase in the *Q*-parameter is recorded (Figure 9b–d), reaching mean values of $Q\approx 6\times 10^{-2}$. This is accompanied by a noisier $G^{\prime }$, G" signal. For samples processed using the conventional screw, C, the increase in nonlinearities varied significantly between measurements, as evidenced in Figure 9b after 11,000 s and in Figure 9c after 1800 s. In contrast, for samples processed with the M-screw, the onset of nonlinearities was observed after approximately 2000 s, limited to around 40%. The contrast is consistent with the dynamic frequency tests, as M-screw samples have a superior distribution of fillers compared to the C-screw samples.

The corresponding dielectric loss $\epsilon^{\prime \prime }$ is plotted as a function of the frequency and time in Figure 10. An electric field applied to a medium will provoke the movement of ‘free’ charges and a local redistribution of ‘fixed’ charges. The mechanism related to the movement of free charges is the conduction, measured by electric conductivity σ*$\left(f_{e}\right)$, and that of fixed charges is polarization, measured by dielectric permittivity ϵ*$\left(f_{e}\right)$. The permittivity is a complex number that varies with the frequency. Pure EBA has two regions of frequency dispersion in the permittivity loss: (1) a broad relaxation peak in the order of MHz, likely caused by polar butyl acrylate groups, and (2) a characteristic low frequency dispersion that likely is a Maxwell–Wagner response due to a barrier blocking conduction process. The two regions of relaxation are clearly defined, but the loss peaks appear to be outside the measured frequency range. Adding nanoparticles to the EBA matrix changes the low frequency dispersion behavior significantly, which could be explained by the nanoparticles affecting the charge transport mechanism and, thus, the net interfacial polarization. It should be noted that the 3D surfaces have been smoothened using an average filter. When at rest, as DES measurements were started prior to the rheological testing, and at the beginning of the deformation process, $\epsilon^{\prime \prime }$ discloses electrical conductivity at low frequencies due to the pre-existing filler network. The initial dielectric signal (t=0 s) appears to be a strong function of the processing conditions with a large variation recorded between the C-screw samples, e.g., compare Figure 10b,c at t=0 s. The highest $\epsilon^{\prime \prime }$ recorded at the beginning of the dynamic time sweep was recorded for the EBA-GnP nanocomposite, in contrast to the results obtained directly via extrusion [3]. This suggests that the combination of extrusion and compression molding creates sufficient anisotropy for electrical percolation compared to the typically highly-oriented morphologies obtained via extrusion. The filler network created by extrusion and compression molding is subsequently destroyed for all samples as the shear deformation progresses. Two subsequent developments are distinguishable, corresponding to the EBA-GnP and hybrid samples, respectively. For EBA-GnP samples, no significant change in $\epsilon^{\prime \prime }$ was recorded in the testing time, similar to the behavior of the rheological nonlinearities. For the hybrid samples, however, the increase in nonlinearities, i.e., the *Q*-parameter, corresponds to an increase in $\epsilon^{\prime \prime }$, signaling the formation of a new electrically-conductive region. The influence of processing conditions stands out through the rheo-dielectric measurements, as well. M-screw samples, albeit exhibiting better dispersion properties compared to C-screw samples, result in a lower dielectric response. This behavior can be explained by the observations of Arino et al. [3] that GnP filler distortion can be responsible for their electric conductivity performance.

**Figure 11:**
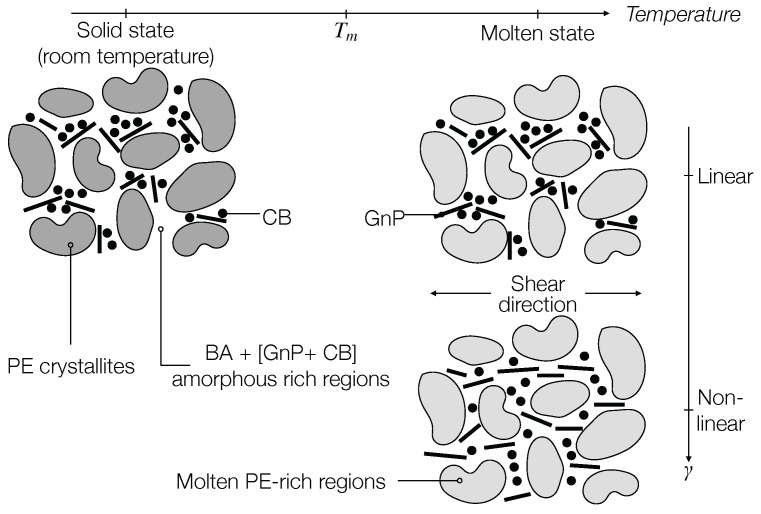
Interpretation of the dynamic behavior of the hybrid nanocomposites studied in the nonlinear viscoelastic regime, based on the model of Leblanc and Jäger [13].

The difference in rheological and dielectric behavior between the sample containing GnP as filler and the hybrid GnP-CB samples can be explained in the framework of the model proposed by Leblanc and Jäger [13]; Figure 11. As nanofillers tend to aggregate in BA regions as opposed to PE regions, when subjected to nonlinear deformations, the initially percolated network is destroyed, and the nanoplatelets become oriented in the flow direction. For EBA-GnP nanocomposites, this means that electrical conductivity can no longer be achieved in the direction perpendicular to the flow direction. In hybrid nanocomposites, however, following the orientation of the GnP, the CB have the ability to efficiently bridge the gaps between the nanoplatelets, being restricted by the BA regions. Thus, a second electrically-conductive network could be created using nonlinear shear deformations.

**Figure 12:**
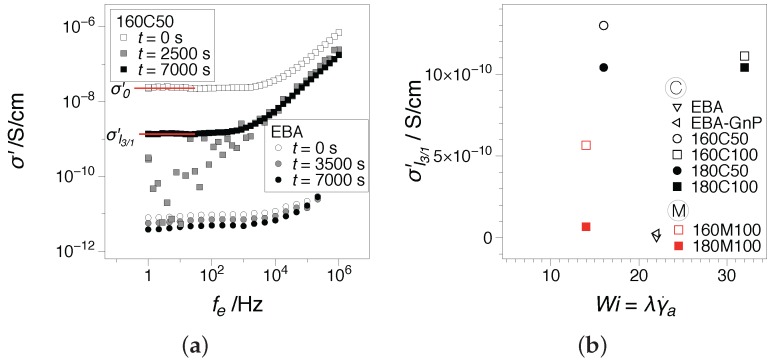
Electrical conductivity as the plateau at low frequencies of the real part of the complex dielectric conductivity [46], $\sigma^{\prime }$, for the samples under investigation: (**a**) examples of the transient dependence and (**b**) conductivity at the onset of $I_{3/1}$ steady variation as a function of the processing (die) Weissenberg number, Wi.

Finally, the electrical conductivity as resulted from rheo-dielectric measurements was considered, as the plateau at low frequencies of the real part of the complex dielectric conductivity [46], $\sigma^{\prime }$; Figure 12a. In the figure, three $\sigma^{\prime }\left(f_{e}\right)$ spectra are reported for the reference EBA and a hybrid. The first spectra is the initial $\sigma^{\prime }$ spectrum recorded before the start of the deformation (see $\sigma_{0}^{\prime }$ at t=0 s). The second is an intermediary value, t=2500 s, while the third corresponds to the conductivity achieved when the rheological nonlinearities reach a quasi-steady variation; see $\sigma_{I_{3/1}}^{\prime }$, t=7000 s for 160C50. The results in Figure 12 capture the transient behavior illustrated in Figure 10. For the hybrid systems, a clear plateau at low frequencies is recorded at the beginning and towards the end of the tests, but not in the intermediate spectrum. By comparison, results for EBA show a slightly decreasing variation, however well within the measurement error, with values two orders of magnitude lower. The electrical conductivity recorded at the end of the experimental testing, $\sigma_{I_{3/1}}^{\prime }$, as a function of the processing Wi number is presented in Figure 12b.

**Figure 13:**
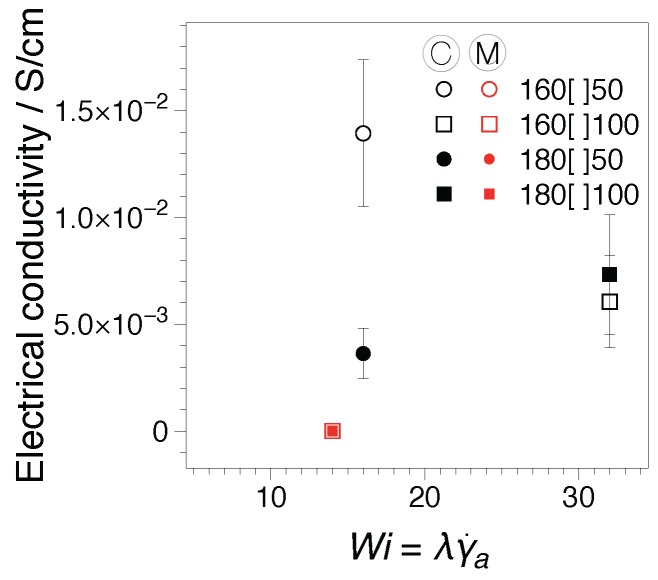
Conductivity in the circuit as a function of the Weissenberg number inside the extrusion die, $Wi=\lambda {\dot{\gamma }}_{a}$, of extruded strands before pelletizing and rheological characterization. Data processed from Arino et al. [3].

For the comparison, the electrical conductivity on extruded strands before being pelletized and compression molded for rheological characterization is shown in Figure 13. The conductivities were measured in a circuit using a two-point method [47] by Arino et al. [3]. Their data have been re-formatted as a dependence of the Weissenberg number, Wi; see Table 1. The two sets of measurements disclose a similar qualitative behavior.

The electrical conductivities of EBA-CB-GnP extruded hybrid nanocomposites was significantly influenced by the applied deformation history, i.e., screw type, and the Wi numbers attained. Given a flow history dominated by shear stresses (C-screw) and at Wi<20, where the orientation of the nanoplatelets is less pronounced, electrical conductivities of ca. 0.01 S/cm were recorded in circuit for extruded samples. More complex deformation histories (M-screw) appeared to generate distorted nanoplatelets [3] with typical conductivities as low as aproximately $10^{-9}$ S/cm regardless of the Wi number. Similarly, the rheo-dielectric measurements point to a maximum in conductivities achieved for Wi<20. In contrast, however, M-screw samples appear to show a better performance in rheo-dielectric measurements compared to the extruded samples. This could suggest that in the framework of the model described by Leblanc and Jäger [13], the flow field-matrix-filler interactions during processing could be further improved.

## 4. Conclusions

The influence of processing conditions on percolated EBA polymers containing GnP and CB was investigated through linear and nonlinear oscillatory shear, extensional and combined rheo-dielectric spectroscopy tests. In linear viscoelastic dynamic frequency sweeps, hybrid EBA-GnP-CB samples processed using a mixing screw (M), composed of a Maillefer region and a Saxton mixer, showed less scattering in their viscosity data compared to samples processes using a conventional screw (C; compression ratio 2:1), at identical filler compositions (5 vol % total filler content, out of which 80 wt % GnP and 20 wt % CB). In dynamic strain sweep tests, the percolation of the samples could be asserted by quantifying the material nonlinear response, $I_{3/1}$, at angular frequencies where a plateau in $G^{\prime }$ is not observable in dynamic frequency sweep tests. The most striking feature of the $I_{3/1}$ dependence on the applied strain amplitude was related to the existence of a plateau at the small amplitude oscillatory shear (SAOS)-medium amplitude oscillatory shear (MAOS) transition. The material response of the EBA matrix showed strain hardening behavior that reveals the branched topology of the polymer. The effect of the processing history on the dispersion properties of the samples was also evidenced through the extensional test and predictions. M-screw samples exhibited higher strain-hardening compared to C-samples. The influence of increasing the volume percent of fillers was also seen as a limit on the strain hardening behavior (9.5 vol %). Otherwise, the strain-hardening behavior of the nanocomposites was preserved irrespective of the processing method. In time-dependent rheo-dielectric tests, there is evidence of dual BA-PE regions, with the fillers mostly concentrated in the BA region. This can be an explanation also of the lower percolation thresholds required in EBA systems compared to other matrix polymers. Thus, in nonlinear measurements, an orientation of the GnP would cause a non-conductive structure, such as is the case of EBA-GnP; however, in the case of the hybrid samples, the CB could be employed to bridge the oriented nano-platelets in order to achieve electrical conductivity perpendicular to the flow direction. The onset on conductivity with time corresponded to an increase in the nonlinear rheological response, as quantified by the *Q*-parameter. Variations in the time-dependent dielectric response could also be attributed to the dispersion properties of the fillers, with C-screw samples showing greater variation in dielectric response. However, lower dielectric responses were achieved for M-screw samples, as evidence of the filler distortion in the case of M-screw samples. When comparing electrical conductivities resulting from the rheo-dielectric measurements to conductivities measured in circuit for extruded samples, a similar qualitative behavior is observed with the highest conductivities recorded for Wi<20. However, M-screw sample conductivities measured in the rheo-dielectric tests suggested that the flow field-matrix-filler interactions during processing could be further improved by the application of alternative processing histories.

## Figures and Tables

**Table 1:** Polymer nanocomposite samples characterized: composition and processing method. The following notations are used: CB, carbon black; GnP, graphite nanoplatelets; $T_{p}$, processing temperature (die); ${\dot{\gamma }}_{a}$, apparent processing shear rate (in the extrusion die) defined in Equation (1); λ, polymer characteristic relaxation time, defined as the inverse of the angular frequency at the crossing between the dynamic moduli; and Wi, the Weissenberg number defined as $Wi={\dot{\gamma }}_{a}\lambda$.

Sample	CB/wt %	GnP/wt %	Screw Type	$T_{p}$/°C	*n*/$s^{-1}$	${\dot{\gamma }}_{a}$/$s^{-1}$	λ/$s^{-1}$	Wi
EBA	-	-	C	160	100	1080	0.02	22
GnP(1)	-	15 (7 vol %)	C	160	50	540	0.04	22
	CB + GnP = 5 vol %							
160C50	20	80	C	160	50	540	0.03	16
160C100	20	80	C	160	100	1080	0.03	32
180C50	20	80	C	180	50	540	0.03	16
180C100	20	80	C	180	100	1080	0.03	32
160M100	20	80	M	160	100	480	0.03	14
180M100	20	80	M	180	100	480	0.03	14

## Abbreviations

The following abbreviations are used in this manuscript:

FT: Fourier-transform
C: compression screw (2:1)
CB: carbon black
DES: dielectric spectroscopy
EBA: ethylene-butyl acrylate
GnP: graphite nanoplatelets
LAOS: large amplitude oscillatory shear
MSF: molecular stress function
M: mixing screw (Maillefer + Saxton mixer)
MWCNT: multi-walled carbon nanotubes
OMMT: organomodified montmorillonite
PCC: precipitated calcium carbonate
PCL: polycaprolactone
PE: polyethylene
SAOS: small amplitude oscillatory shear
SWCNT: single-walled carbon nanotubes

## References

[1] Ferrari A.C., Bonaccorso F., Fal’ko V., Novoselov K.S., Roche S., Boggild P., Borini S., Koppens F.H.L., Palermo V., Pugno N. (2015). Science and technology roadmap for graphene, related two-dimensional crystals, and hybrid systems. Nanoscale.

[2] Oxfall H., Ariu G., Gkourmpis T., Rychwalski R., Rigdahl M. (2015). Effect of carbon black on electrical and rheological properties of graphite nanoplatelets/poly(ethylene-butyl acrylate) composites. Express Polym. Lett..

[3] Arino R., Diez E.A., Rigdahl M. (2016). Enhancing the electrical conductivity of carbon black/graphite nanoplatelets: Poly(ethylene-butyl acrylate) composites by melt extrusion. J. Appl. Polym. Sci..

[4] Induchoodan G., Kádár R. (2016). Tailoring polymer nanocomposite microstructure by controlling orientation, dispersion and exfoliation of GnP in LDPE via extrusion flow. Trans. Nord. Rheol. Soc..

[5] Song Y., Zheng Q. (2016). Concepts and conflicts in nanoparticles reinforcement to polymers beyond hydrodynamics. Prog. Mater. Sci..

[6] Cassagnau P. (2013). Linear viscoelasticity and dynamics of suspensions and molten polymers filled with nanoparticles of different aspect ratios. Polymer.

[7] Kim H., Abdala A.A., Macosko C.W. (2010). Graphene/Polymer Nanocomposites. Macromolecules.

[8] Zhao J., Morgan A.B., Harris J.D. (2005). Rheological characterization of polystyrene–clay nanocomposites to compare the degree of exfoliation and dispersion. Polymer.

[9] Wagener R., Reisinger T.J. (2003). A rheological method to compare the degree of exfoliation of nanocomposites. Polymer.

[10] Aranguren M.I., Mora E., DeGroot J.V., Macosko C.W. (1992). Effect of reinforcing fillers on the rheology of polymer melts. J. Rheol..

[11] Hassanabadi H.M., Wilhelm M., Rodrigue D. (2014). A rheological criterion to determine the percolation threshold in polymer nano-composites. Rheol. Acta.

[12] Hassanabadi H.M., Abbasi M., Wilhelm M., Rodrigue D. (2013). Validity of the modified molecular stress function theory to predict the rheological properties of polymer nanocomposites. J. Rheol..

[13] Leblanc J.L., Jäger K.M. (2016). Investigating Nonlinear Viscoelastic Properties of Molten Carbon Black/Poly(ethylene-*co*-butyl acrylate) Composites, Using Fourier Transform Rheometry and Other Test Techniques. J. Appl. Polym. Sci..

[14] Ahirwal D., Palza H., Schlatter G., Wilhelm M. (2014). New way to characterize the percolation threshold of polyethylene and carbon nanotube polymer composites using Fourier transform (FT) rheology. Korea-Aust. Rheol. J..

[15] Lim H.T., Ahn K.H., Hong J.S., Hyun K. (2013). Nonlinear viscoelasticity of polymer nanocomposites under large amplitude oscillatory shear flow. J. Rheol..

[16] Nelson J.K., Hu Y. (2005). Nanocomposite dielectrics—Properties and implications. J. Phys. D.

[17] Jeong K.U., Lim J.Y., Lee J.Y., Kang S.L., Nah C. (2010). Polymer nanocomposites reinforced with multi-walled carbon nanotubes for semiconducting layers of high-voltage power cables. Polym. Int..

[18] Oxfall H. (2013). Manufacturing and Characterization of Filled Polymeric Systems. Ph.D. Thesis.

[19] Sinha Ray S., Okamoto M. (2003). Polymer/layered silicate nanocomposites: A review from preparation to processing. Prog. Polym. Sci..

[20] Kremer F., Schönhals A. (2003). Broadband Dielectric Spectroscopy.

[21] Potschke P., Abdel-Goad M., Alig I., Dudkin S., Lellinger D. (2004). Rheological and dielectrical characterization of melt mixed polycarbonate-multiwalled carbon nanotube composites. Polymer.

[22] Alig I., Skipa T., Lellinger D., Potschke P. (2008). Destruction and formation of a carbon nanotube network in polymer melts: Rheology and conductivity spectroscopy. Polymer.

[23] Moreira L., Fulchiron R., Seytre G., Dubois P., Cassagnau P. (2010). Aggregation of Carbon Nanotubes in Semidilute Suspension. Macromolecules.

[24] Bharati A., Wubbenhorst M., Moldenaers P., Cardinaels R. (2016). Effect of Compatibilization on Interfacial Polarization and Intrinsic Length Scales in Biphasic Polymer Blends of PαMSAN and PMMA: A Combined Experimental and Modeling Dielectric Study. Macromolecules.

[25] Figuli R., Schwab L., Lacayo-Pineda J., Deckmann H., Wilhelm M. (2016). Combined Dielectric (DEA) and Dynamic Mechanical Thermal Analysis (DMTA) in Compression Mode.

[26] Costa L., Achour M., Graça M., Hasnaoui M.E., Outzourhit A., Oueriagli A. (2010). Dielectric properties of the ethylene butylacrylate/carbon black nanocomposites. J. Non-Cryst. Solids.

[27] Kádár R., Naue I.F., Wilhelm M. (2016). First normal stress difference and in-situ spectral dynamics in a high sensitivity extrusion die for capillary rheometry via the ‘hole effect’. Polymer.

[28] Merger D., Wilhelm M. (2014). Intrinsic nonlinearity from LAOStrain—Experiments on various strain- and stress-controlled rheometers: a quantitative comparison. Rheol. Acta.

[29] Hyun K., Wilhelm M., Klein C., Cho K.S., Nam J., Ahn K., Lee S., Ewoldt R., McKinley G. (2011). A review of nonlinear oscillatory shear tests: Analysis and application of large amplitude oscillatory shear (LAOS). Prog. Polym. Sci..

[30] Walters K. (1975). Rheometry.

[31] Calin A., Wilhelm M., Balan C. (2010). Determination of the non-linear parameter (mobility factor) of the Giesekus constitutive model using {LAOS} procedure. J. Non-Newton. Fluid Mech..

[32] Cziep M.A., Abbasi M., Heck M., Arens L., Wilhelm M. (2016). Effect of Molecular Weight, Polydispersity, and Monomer of Linear Homopolymer Melts on the Intrinsic Mechanical Nonlinearity $3_{0}^{Q}$(ω) in MAOS. Macromolecules.

[33] Hyun K., Wilhelm M. (2009). Establishing a New Mechanical Nonlinear Coefficient *Q* from FT-Rheology: First Investigation of Entangled Linear and Comb Polymer Model Systems. Macromolecules.

[34] Ewoldt R.H., Hosoi A.E., McKinley G.H. (2008). New measures for characterizing nonlinear viscoelasticity in large amplitude oscillatory shear. J. Rheol..

[35] Rolón-Garrido V.H. (2014). The molecular stress function (MSF) model in rheology. Rheol. Acta.

[36] Abbasi M., Golshan Ebrahimi N., Nadali M., Khabazian Esfahani M. (2012). Elongational viscosity of LDPE with various structures: Employing a new evolution equation in MSF theory. Rheol. Acta.

[37] Abbasi M., Golshan Ebrahimi N., Wilhelm M. (2013). Investigation of the rheological behavior of industrial tubular and autoclave LDPEs under SAOS, LAOS, transient shear, and elongational flows compared with predictions from the MSF theory. J. Rheol..

[38] Polizos G., Tuncer E., Tomer V., Sauers I., Randall C., Manias E. (2013). Dielectric Spectroscopy of Polymer-Based Nanocomposite Dielectrics with Tailored Interfaces and Structured Spatial Distribution of Fillers. Nanoscale Spectroscopy with Applications.

[39] Meins T., Hyun K., Ratzsch K., Friedrich C., Struth B., Wilhelm M. (2011). Combined methods in Rheology: Rheo-SAXS, Rheo-NMR and Rheo-Dielectric to bridge length and time scales. Annu. Trans. Nord. Rheol. Soc..

[40] Meins T., Dingenouts N., Kübel J., Wilhelm M. (2012). In Situ Rheodielectric, ex Situ 2D-SAXS, and Fourier Transform Rheology Investigations of the Shear-Induced Alignment of Poly(styrene-*b*-1,4-isoprene) Diblock Copolymer Melts. Macromolecules.

[41] Hyun K., Höfl S., Kahle S., Wilhelm M. (2009). Polymer motion as detected via dielectric spectra of 1,4-*cis*-polyisoprene under large amplitude oscillatory shear (LAOS). J. Non-Newton. Fluid Mech..

[42] Rolón-Garrido V., Wagner M. (2007). The MSF model: Relation of nonlinear parameters to molecular structure of long-chain branched polymer melts. Rheol. Acta.

[43] Chan Y., White J.L., Oyanagi Y. (1978). A Fundamental Study of the Rheological Properties of Glass-Fiber-Reinforced Polyethylene and Polystyrene Melts. J. Rheol..

[44] Takahashi T., Wu W., Toda H., Takimoto J.I., Akatsuka T., Koyama K. (1997). Elongational viscosity of ABS polymer melts with soft or hard butadiene particles. J. Non-Newton. Fluid Mech..

[45] Li L., Masuda T., Takahashi M. (1990). Elongational flow behavior of ABS polymer melts. J. Rheol..

[46] Schopp S., Thomann R., Ratzsch K.F., Kerling S., Altstädt V., Mülhaupt R. (2014). Functionalized Graphene and Carbon Materials as Components of Styrene-Butadiene Rubber Nanocomposites Prepared by Aqueous Dispersion Blending. Macromol. Mater. Eng..

[47] Heaney M.B. (2003). Electrical Measurement, Signal Processing, and Displays.

